# Association Between Serum Uric Acid Levels and Oxido-Inflammatory Biomarkers With Coronary Artery Disease in Type 2 Diabetic Patients

**DOI:** 10.7759/cureus.47913

**Published:** 2023-10-29

**Authors:** Amal F Gharib, Ola E Nafea, Amani A Alrehaili, Abdulraheem Almalki, Afaf Alharthi, Ohud Alsalmi, Fouzeyyah A Alsaeedi, Ayman Alhazmi, Mamdouh Allahyani, Rasha L Etewa, Alaa H Alsulimani, Sara O Badr

**Affiliations:** 1 Department of Clinical Laboratory Sciences, College of Applied Medical Sciences, Taif University, Taif, SAU; 2 Department of Clinical Pharmacy, College of Pharmacy, Taif University, Taif, SAU; 3 Department of Pathology, College of Medicine, Jouf University, Sakaka, SAU; 4 Department of Laboratory, King Faisal Medical Complex (KFMC) and Research Center, Taif, SAU; 5 Department of Internal Medicine, Faculty of Medicine, Port Said University, Port Said, EGY

**Keywords:** uric acid, type 2 diabetes mellitus, oxidative injury, inflammation, coronary artery disease

## Abstract

Background: Cardiovascular disease signifies a major cause of morbidity and mortality among patients with type 2 diabetes mellitus (T2DM). Serum uric acid (SUA) levels are elevated during the initial phases of impaired glucose metabolism. This work was designed to explore the association between SUA levels, serum oxido-inflammatory biomarkers, and the risk of coronary artery disease (CAD) in T2DM patients as the primary outcome. The secondary outcome was to assess the prognostic role of SUA in the prediction of the risk of CAD in T2DM patients.

Methods: In this case-control study, we enrolled 110 patients with T2DM who were further divided into patients with CAD and without CAD. In addition, 55 control participants were stringently matched to cases by age.

Results: Diabetic patients with CAD had significantly higher serum levels of the inflammatory biomarkers and the oxidative malondialdehyde but significantly lower levels of serum total antioxidant capacity (TAC) compared with the controls and diabetic patients without CAD. Significant positive correlations existed between SUA levels and serum levels of the inflammatory biomarkers and malondialdehyde, while a significant negative correlation existed between SUA levels and serum TAC. SUA demonstrated an accepted discrimination ability. SUA can differentiate between T2DM patients with CAD and patients without CAD, an area under the curve of 0.759.

Conclusions: Elevated serum levels of SUA and oxido-inflammatory biomarkers are associated with an increased risk of CAD in T2DM. SUA levels reflect the body's inflammatory status and oxidant injury in T2DM. SUA could be utilized as a simple biomarker in the prediction of CAD risk in T2DM.

## Introduction

Diabetes mellitus (DM) is a significant disease of our modern era, and its prevalence is estimated to increase further in the coming years. According to the International Diabetes Federation (IDF), by 2045, the global prevalence of adult-onset DM will reach 693 million individuals. This statistic emphasizes the need for awareness and proactive measures to combat this trend [1].

DM is a chronic metabolic disease that causes high blood sugar levels due to either a lack of insulin or insulin resistance in peripheral tissues [2]. Type 2 diabetes mellitus (T2DM) is a rapidly growing global pandemic that has become a major concern for healthcare professionals worldwide. T2DM can lead to various complications that affect both the micro-vascular and macro-vascular systems [3].

Individuals with T2DM are at a higher risk of suffering from cardiovascular disease, which can lead to severe morbidity and mortality. Studies show diabetic patients are two to four times more likely to experience cardiovascular events than non-diabetic individuals, especially when their glycemic control is inadequate [4].

Diabetic patients have a higher likelihood of developing atherosclerotic coronary artery disease (CAD) due to various factors, including metabolic conditions such as hyperglycemia, dyslipidemia, and insulin resistance. These factors contribute to dysfunction in endothelial cells and vascular smooth muscles, impaired platelet function, and abnormal coagulation [5].

CAD is the third-leading cause of death globally, claiming an estimated 17.8 million lives annually. Although avoidable, CAD is still a significant cause of premature death and disability [6]. Oxidative injury and inflammation are critical contributors to cardio-metabolic disorders. Additionally, persistent hyperglycemia promotes the release of free radicals, leading to chronic subclinical inflammation and vascular dysfunction [7-9].

A strong association exists between hyperglycemia, oxidative injury, inflammation, and T2DM development and progression. Moreover, oxidative injury elicits the production of inflammatory mediators and inflammatory cascades, enhancing the generation of reactive oxygen species. In addition, human insulin resistance involves adipose tissue derangement, lipotoxicity, glucotoxicity, oxidative injury, and subclinical inflammation [10,11]. 

In humans, uric acid (UA) serves as the end product of purine nucleotide metabolism. The equilibrium of UA levels in the body is maintained through a delicate interplay between its production and elimination via the kidneys and intestines. The kidneys are significant regulators of circulating uric acid levels. Approximately two-thirds of UA is excreted in the kidneys, and the remaining one-third is excreted into the intestines [12-14]. UA is a potent endogenous antioxidant, but excessive levels of UA, or hyperuricemia, have been linked to oxidative stress, inflammation, and endothelial vascular damage. A high UA level is associated with various pathological conditions such as gout, chronic kidney disease, hypertension, atherosclerosis, CAD, and cardiac failure. In diabetic patients, an elevated serum uric acid (SUA) level is linked to the development of T2DM. During the initial stages of impaired glucose metabolism, SUA levels tend to increase. Hyperuricemia is also associated with micro- and macrovascular complications among diabetic patients [15-18].

It is possible that high levels of SUA can be an indicator of the likelihood of developing T2DM. Research has shown that there may be a connection between high SUA levels and impaired glucose metabolism during the early stages of T2DM [19].

In the current study, we hypothesized that increased SUA levels and increased serum levels of oxido-inflammatory biomarkers are associated with an increased risk of CAD in patients with T2DM. Consequently, this study was conducted to explore the association between SUA levels, serum oxido-inflammatory biomarkers, such as tumor necrosis factor-alpha (TNF-α), interleukin 6 (IL-6), C-reactive protein (CRP), total antioxidant capacity (TAC), and malondialdehyde (MDA), and the risk of CAD among patients with T2DM as the primary outcome. At the same time, the secondary outcome was to assess the prognostic role of SUA in predicting the risk of CAD in patients with T2DM.

## Materials and methods

Subjects and study setting

This was a case-control study. Patients were randomly selected from the Cardiology Outpatient Clinic at King Faisal Medical Complex in Taif, Saudi Arabia, between December 2022 and May 2023. One hundred and ten T2DM patients (67 men and 43 women) were recruited, and their ages ranged from 50 to 78 years. The eligibility criteria included patients with T2DM, diagnosed according to the American Diabetes Association in 2014, from both sexes. According to the coronary angiographic findings, electrocardiogram and cardiac catheterization findings, diabetic patients were further divided into two groups: patients with CAD and patients without CAD. ﻿The exclusion criteria included the following: (1) Diseases that affect SUA levels, such as cardiac diseases, hepatic diseases, chronic renal diseases, gout, and cancer. (2) Medications or vitamin supplements that affect SUA levels, such as salicylates, allopurinol, probenecid, ascorbic, folic acid, niacin, febuxostat, and diuretics. (3) Use of analgesics and antioxidant therapy in the past three months. (4) Type 1 diabetes, recent myocardial infarction, coronary revascularization, infections, chronic inflammatory disorders, and autoimmune diseases [20]. ﻿In addition, 55 apparently healthy subjects were randomly selected and served as the control group. Control participants were excluded if they were taking vitamin supplements or antioxidants. ﻿They were undergoing routine checkups or routine preoperative testing for elective minor surgical procedures. All eligible participants underwent detailed history-taking, a complete physical examination, and relevant biochemical analyses.

Sample size calculation

The sample size was 165 participants (55 healthy participants, 55 T1D patients without CAD, and 55 T1D patients with CAD) at a mean difference between two independent means (SUA values in the control group versus mild CAD group, 5.3±1.5 vs 6.2±1.6 mg/dl, respectively) according to the study of Ekici et al. [17] at a power of 85% and a type I error threshold (α) <0.05. G*power 3.1 was used to calculate the sample size [21].

Blood sampling and biochemical analyses

Ten-milliliter peripheral venous blood samples, after overnight fasting, were drawn from all participants and divided into three tubes: ethylenediaminetetraacetic acid (EDTA) tubes for estimation of the glycated hemoglobin (HbA1C) content using an automated glycosylated hemoglobin analyzer, sodium fluoride tubes for measurement of fasting blood glucose (FBG) levels according to the glucose oxidase method as described by Trinder [22], and plain tubes that left until clotting and centrifuged at 3000 rpm for 15 min. Then serum samples were used to analyze the SUA and serum lipids (triglycerides (TG), total cholesterol (TC), high-density lipoprotein cholesterol (HDL-C), and low-density lipoprotein cholesterol (LDL-C)) using enzymatic colorimetric techniques and oxido-inflammatory biomarkers (TNF-α, IL6, CRP, TAC, and MDA) using commercially available enzyme-linked immunosorbent assay (ELISA) kits.

Statistics

Normally distributed continuous variables were presented as the mean±standard deviation (SD). While non-normally distributed continuous variables were presented as median (25th to 75th percentiles). The Kolmogorov-Smirnov test was used to check the assumption of normality of distribution. Leven's test was used to check the assumption of homogeneity of variance. Categorical variables were presented by frequency and percentage. The chi-squared test was used to determine the relationship between two categorical variables. The one-way analysis of variance (ANOVA) was used to determine the significant differences between the means of two or more independent groups on a continuous dependent variable. Welch’s ANOVA test was used if equal variances were violated. Post-hoc tests following the ANOVA test (Tukey test if equal variances were assumed; Tamhane’s T2 test if equal variances were violated) were used for multiple comparisons among groups. The Kruskal-Wallis H test is a non-parametric alternative to the one-way ANOVA. Dunn’s is a post-hoc test following the Kruskal-Wallis H test. Pearson’s correlation coefficient was used to test the strength of the linear relationship between two continuous variables; at least one of the two variables must follow a normal distribution. The receiver operating characteristic (ROC) curve was used to determine the prognostic performance of SUA levels in the prediction of CAD among diabetic patients. Differences were considered significant at P<0.05. All statistical comparisons were two-tailed. Statistical analyses were performed using Statistical Package of Social Science (SPSS) version 25.0 (IBM; Armonk, New York, USA). The correlation matrix was plotted by the R statistical package (ggplot2 and corrplot) version 3.2.3 (www.r-project.org/).

## Results

**Figure 1:**
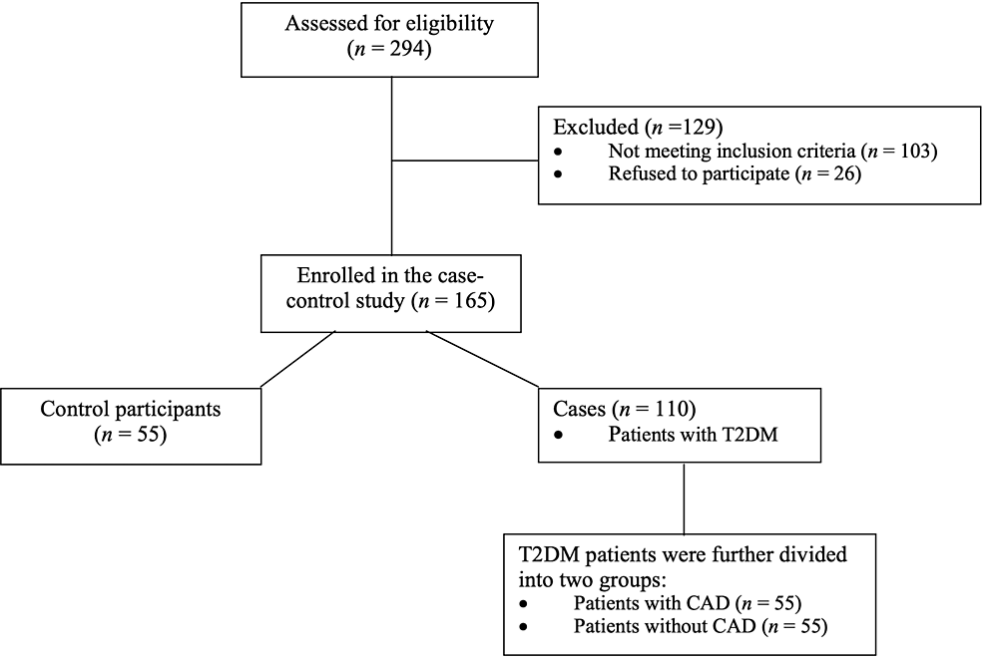
Flowchart of the study. T2DM: Type 2 diabetes mellitus; CAD: coronary artery disease.

Figure 1 represents the flowchart of the study. Both healthy participants’ and patients’ baseline characteristics, the laboratory findings (blood glucose indices, lipid profiles, and SUA levels), as well as the oxido-inflammatory biomarkers’ results, were stratified by SUA levels’ tertile into low, intermediate, and high.

Baseline characteristics of the healthy participants and patients with T2DM

A total of 55 healthy participants and 110 patients with T2DM were enrolled in this study. The mean age was 63.4±8.0 years, 65.0±8.1 years, and 62.4±6.8 years in the control, non-CAD, and CAD patients, respectively. Most patients with CAD were current smokers (65.5%, 36 patients) and hypertensive (70.9%, 39 patients). Other baseline features of the study participants are represented in Table 1. Compared with diabetic patients with low SUA levels, those with high SUA levels had a statistically significantly higher body mass index (p<0.001). Significantly more patients with high SUA levels had CAD than patients with intermediate or low SUA levels (75.7%, 28 patients) versus (44.7%, 17 patients, and 28.6%, 10 patients, respectively) (P<0.001) (Table 2).

**Table 1:** Baseline characteristics of the healthy participants and diabetic patients. Data are mean±standard deviation, unless otherwise mentioned. Significant differences at P-value<0.001. †Chi-squared test. ‡One-way analysis of variance (ANOVA) test. ﻿Means in a row without common superscript letters significantly differ (P<0.05) by a one-way ANOVA followed by post-hoc Tukey’s multiple comparison test. CAD: coronary artery disease; T2DM: type 2 diabetes mellitus.

Baseline characteristics	Healthy participants	T2DM patients		P-value
		No-CAD	CAD	
	n=55	n=55	n=55	
Male sex, n (%)	35 (63.6)	33 (60)	34 (61.8)	0.93†
Age (years)	63.4±8.0^a^	65.0±8.1^a^	62.4±6.8^a^	0.21‡
Current smoking, n(%)	10 (18.2)	24 (43.6)	36 (65.5)	<0.001†
Hypertension, n(%)	0 (0)	32 (58.2)	39 (70.9)	<0.001†
Body mass index (kg/m^2^)	24.8±3.6^a^	26.5±4.1^a^	28.5±4.7^b^	<0.001‡

**Table 2:** Baseline characteristics stratified according to SUA levels of the diabetic patients. Data are mean±standard deviation, unless otherwise mentioned. Significant differences at P-value<0.05. †Chi-squared test. ‡One-way analysis of variance (ANOVA) test. ﻿Means in a row without common superscript letters significantly differ (P<0.05) by a one-way ANOVA followed by post-hoc Tukey’s multiple comparison test. CAD: coronary artery disease; SUA: serum uric acid.

Baseline characteristics	SUA levels			P-value
	Low (<5.39 mg/dl)	Intermediate (5.39-7.55 mg/dl)	High (7.56+ mg/dl)	
	n=35	n=38	n=37	
Male sex, n(%)	22(62.9)	25 (65.8)	20 (54.1)	0.56†
Age (years)	65.1±7.6	62.7±8.0	63.5±5	0.39‡
Current smoking, n(%)	17 (48.6)	21 (55.3)	22 (59.5)	0.65†
Hypertension, n(%)	19 (54.3)	23 (60.5)	29 (78.4)	0.083†
Body mass index (kg/m^2^)	26.5±4.5^a^	26.9±4.3^a,b^	29.2±4.4^b^	0.023‡
CAD, n(%)	10 (28.6)	17 (44.7)	28 (75.7)	<0.001†

Laboratory findings (blood glucose indices, lipid profiles, and SUA levels) of the healthy participants and patients with T2DM

Blood glucose indices (FBG and HbA1c), lipid profile parameters (TG, TC, and LDL-C), and SUA levels were statistically significantly higher in the diabetic patients with CAD compared with the healthy participants and diabetic patients without CAD (P<0.001, each). While serum HDL-C levels were statistically significantly lower in the diabetic patients with CAD (40.3) than the healthy participants (64.7) and diabetic patients without CAD (55.9) (P<0.001, each) (Table 3). Diabetic patients with high SUA levels had statistically significantly higher levels of FBG (215.7±62.6) and TC (260.7) than patients with intermediate (166.4±55.9), (242) or low SUA levels (144.9±55.6), (239.3) (P≤0.001, each). While diabetic patients with high SUA levels had statistically significantly higher LDL-C (205.4) but lower HDL-C (43.4±8.8) compared with those with low SUA levels (143.7), (50.4±10.3) (P=0.009 and P=0.011, respectively) (Table 4).

**Table 3:** Laboratory findings of the healthy participants and diabetic patients. Data are mean±standard deviation or median (25th to 75th percentiles). Significant differences at P-value<0.001. †One-way analysis of variance (ANOVA) test. ﻿‡Kruskal-Wallis H test. ﻿Means or medians in a row without common superscript letters significantly differ (P<0.05) by a one-way ANOVA followed by post-hoc Tukey’s multiple comparison test or Kruskal-Wallis H test followed by ﻿Dunn’s post-hoc multiple comparison test, respectively. CAD: coronary artery disease; FBG: fasting blood glucose; HbA1c: hemoglobin A1c; HDL-C: high-density lipoprotein (HDL) cholesterol; LDL-C: low-density lipoprotein (LDL) cholesterol; T2DM: type 2 diabetes mellitus, TC: total cholesterol; TG: triglycerides.

Laboratory findings	Reference range	Healthy participants	T2DM patients		P-value
			No-CAD	CAD	
		n=55	n=55	n=55	
FBG (mg/dl)	70-100	89.3±20.9^a^	121.2±28.7^b^	231.1±38.7^c^	<0.001†
HbA1c%	4–5.6	5.0±1.02^a^	6.4±1.4^b^	8.0±1.8^c^	<0.001†
TG (mg/dl)	150-199	128 (118.3-135.7)^a^	197.4 (188.5-214.7)^a^	242.6 (227-252.5)^c^	<0.001‡
TC (mg/dl)	125-200	150.6 (135.4-161.2)^a^	230.5 (22.5-240.5)^b^	270.7 (253.7-240.5)^c^	<0.001‡
LDL-C (mg/dl)	100-129	57.7 (49.9-63.7)^a^	139.5 (129.5-148.3)^b^	234.7 (199.9-243.6)^c^	<0.001‡
HDL-C (mg/dl)	35-65	64.7 (59.9-70.5)^a^	55.9 (43.8-61.9)^b^	40.3 (35.0-46.8)^c^	<0.001‡
Uric acid (mg/dl)	3.5-7.2	4.0±1.5^a^	5.5±1.4^b^	7.7±2.0^c^	<0.001†

**Table 4:** Laboratory findings stratified according to SUA levels of the diabetic patients. Data are mean±SD or median (25th to 75th percentiles). Significant differences at P-value<0.05. †One-way analysis of variance (ANOVA) test. ﻿‡Kruskal-Wallis H test. ﻿Means or medians in a row without common superscript letters significantly differ (P<0.05) by a one-way ANOVA followed by post-hoc Tukey’s multiple comparison test or Kruskal-Wallis H test followed by ﻿Dunn’s post-hoc multiple comparison test, respectively. FBG: fasting blood glucose; HbA1c: hemoglobin A1c; HDL-C: high-density lipoprotein (HDL) cholesterol; LDL-C: low-density lipoprotein (LDL) cholesterol; SUA: serum uric acid; T2DM: type 2 diabetes mellitus, TC: total cholesterol; TG: triglycerides.

Laboratory findings	Reference range	SUA levels			P-value
		Low (<5.39 mg/dl)	Intermediate (5.39-7.55 mg/dl)	High (7.56+ mg/dl)	
		n=35	n=38	n=37	
FBG (mg/dl)	70-100	144.9±55.6^a^	166.4±55.9^a^	215.7±62.6^b^	<0.001†
HbA1c%	4–5.6	6.9±1.6	7.2±1.4	7.6±1.9	0.17†
TG (mg/dl)	150-199	210.0 (193.4-220.6)	218.1 (189.6-238)	235.4 (200.7-251.4)	0.050‡
TC (mg/dl)	125-200	239.3 (225.5-258.8)^a^	242 (140.1-233.8)^a^	260.7 (242.8-292.2)^b^	0.002‡
LDL-C (mg/dl)	100-129	143.7 (130.9-208.7)^a^	155.7 (140.1-233.8)^a,b^	205.4 (167.8-241.2)^b^	0.009‡
HDL-C (mg/dl)	35-65	50.4±10.3^a^	47.9±10.6^ a,b^	43.4±8.8^b^	0.011†

Oxido-inflammatory biomarkers’ results of the healthy participants and patients with T2DM

Serum levels of the inflammatory biomarkers (TNF-α, IL6, and CRP) were statistically significantly higher in the diabetic patients with CAD (57.9±9.0, 49.9, and 7.9) compared with the healthy participants (3.7±1.4, 3.7, and 2.1) and diabetic patients without CAD (32.4±6.5, 28.7, and 6.3) (P≤0.001, each). In addition, serum TAC levels were statistically significantly lower in the diabetic patients with CAD (0.9) than in the healthy participants (2.2) and diabetic patients without CAD (1.7) (P≤0.001, each). While serum levels of the oxidative MDA were statistically significantly higher in the diabetic patients with CAD (9.0) than in the healthy participants (4.5) and diabetic patients without CAD (7.5) (P≤0.001, each) (Table 5). In comparison to diabetic patients with low SUA levels, those with high SUA levels showed significantly elevated levels of the inflammatory markers TNF-α (35.5, 51.5) and IL6 (31.8±12.2, 42.8±13.4) (P=0.004 and P=0.002, respectively).

Furthermore, diabetic patients with high SUA levels had statistically significantly higher CRP levels (7.8±1.4) than patients with intermediate (6.8±1.4) or low SUA levels (6.4±1.4) (P=0.007 and P≤0.001, respectively). In addition, serum levels of the oxidative marker MDA were significantly higher in patients with high (9.3±1.6) and intermediate (8.6±1.5) levels of SUA compared to patients with low SUA levels (7.3±1.1) (P≤0.001, each) (Table 6).

**Table 5:** Oxido-inflammatory biomarkers of the healthy participants and diabetic patients. Data are mean±standard deviation or median (25th to 75th percentiles). Significant differences at P-value<0.001. †One-way Welch’s analysis of variance (ANOVA) test. ﻿‡Kruskal-Wallis H test. Means or medians in a row without common superscript letters significantly differ (P<0.05) by a one-way Welch’s ANOVA followed by a post-hoc Tamhane’s T2 multiple comparison test or Kruskal-Wallis H test followed by Dunn’s post-hoc multiple comparison test, respectively. CAD: coronary artery disease; CRP: c-reactive protein; IL6: interleukin 6; MDA: malondialdehyde; T2DM: type 2 diabetes mellitus; TAC: total antioxidant capacity; TNF-α: tumor necrosis factor-alpha.

Oxido-inflammatory biomarkers	Reference range	Healthy participants	T2DM patients		P-value
			No-CAD	CAD	
		n=55	n=55	n=55	
TNF-α (pg/ml)	0.0-20	3.7±1.4^a^	32.4±6.5^b^	57.9±9.0^c^	<0.001†
IL6 (pg/ml)	0.0-12	3.7 (2.7-4.9)^a^	28.7 (20.9-33.7)^b^	49.9 (41.9-54.7)^c^	<0.001‡
CRP (mg/L)	<3	2.1 (0.9-3.1)^a^	6.3 (5.1-7.0)^b^	7.9 (6.8-8.7)^c^	<0.001‡
TAC (mmol/L)	0.05-5	2.2 (1.6-3.0)^a^	1.7 (0.9-2.4)^a^	0.9 (0.5-1.7)^b^	<0.001‡
MDA (nmol/mL)	2.02-4.65	4.5 (3.5-6.0)^a^	7.5 (6.5-8.9)^b^	9.0 (7.8-10.5)^c^	<0.001‡

**Table 6:** Oxido-inflammatory biomarkers stratified according to SUA levels of the diabetic patients. Data are mean±standard deviation or median (25th to 75th percentiles). Significant differences at P-value<0.001. ﻿†Kruskal-Wallis H test. ‡One-way Welch’s analysis of variance (ANOVA) test. Means or medians in a row without common superscript letters significantly differ (P<0.05) by a one-way Welch’s ANOVA followed by a post-hoc Tamhane’s T2 multiple comparison test or Kruskal-Wallis H test followed by Dunn’s post-hoc multiple comparison test, respectively. CRP: c-reactive protein; IL6: interleukin 6; MDA: malondialdehyde; SUA: serum uric acid; T2DM: type 2 diabetes mellitus; TAC: total antioxidant capacity; TNF-α: tumor necrosis factor-alpha.

Oxido-inflammatory biomarkers	Reference range	SUA levels			P-value
		Low (<5.39 mg/dl)	Intermediate (5.39-7.55 mg/dl)	High (7.56+ mg/dl)	
		n=35	n=38	n=37	
TNF-α (pg/ml)	0.0-20	35.5 (26.9-53.6)^a^	42.4 (32.9-57.5)^a,b^	51.5 (38-64.1)^b^	0.004†
IL6 (pg/ml)	0.0-12	31.8±12.2^a^	38.3±13.0^a,b^	42.8±13.4^b^	0.002‡
CRP (mg/L)	<3	6.4±1.4^a^	6.8±1.4^a^	7.8±1.4^b^	<0.001‡
TAC (mmol/L)	0.05-5	1.6 (1.0-2.2)	1.2 (0.6-1.8)	0.8 (0.4-1.8)	0.052†
MDA (nmol/mL)	2.02-4.65	7.3±1.1^a^	8.6±1.5^b^	9.3±1.6^b^	<0.001‡

**Figure 2:**
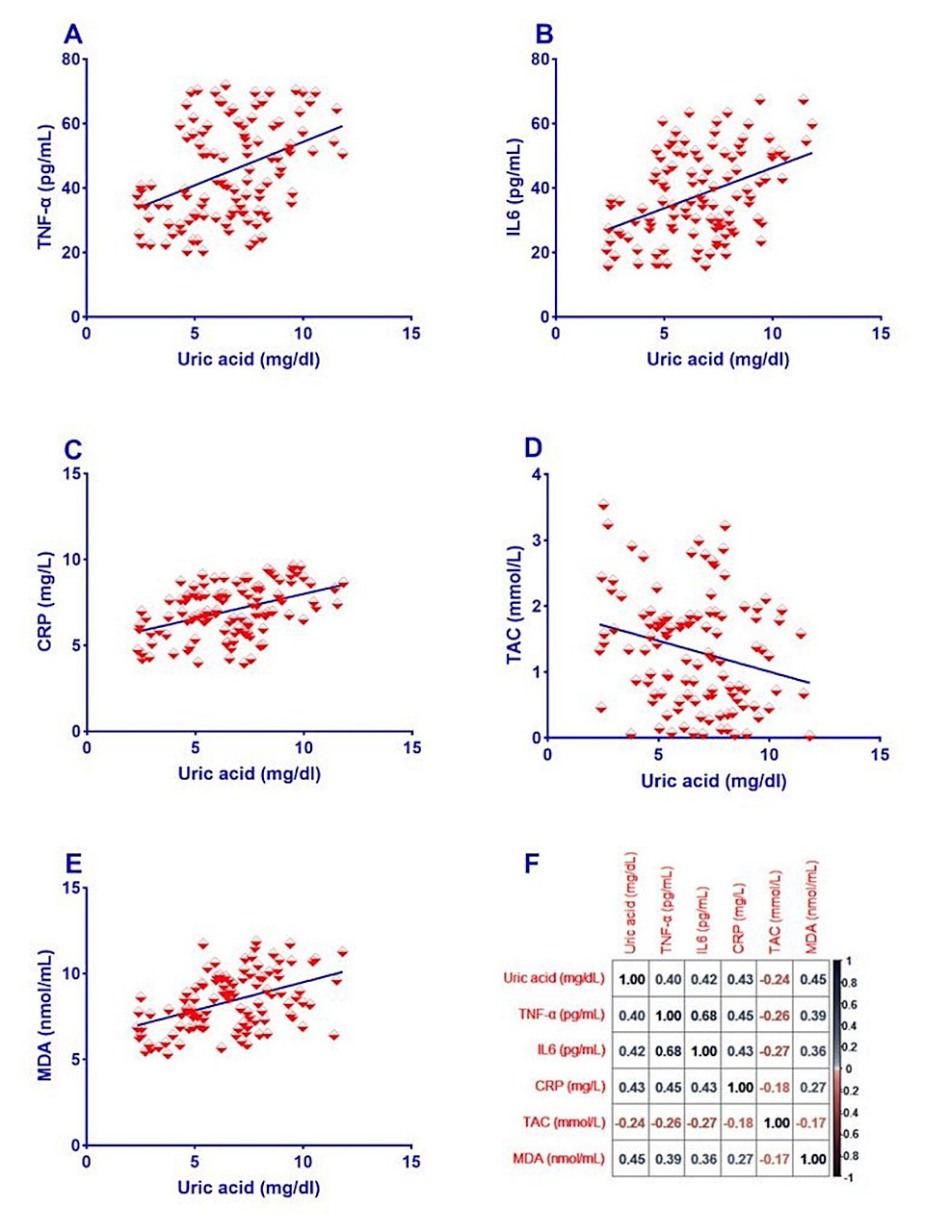
Correlation between oxido-inflammatory biomarkers and SUA levels of patients with T2DM. Among T2DM patients, SUA levels had a positive correlation with the serum levels of the inflammatory biomarkers (TNF-α, IL6, and CRP) and oxidative MDA (P<0.001, each). Furthermore, among T2DM patients, there was a statistically significant negative connection (P=0.010) between SUA levels and serum TAC levels. Panel A: Correlation between serum TNF-α and SUA levels of patients with T2DM. Panel B: Correlation between serum IL6 and SUA levels of patients with T2DM. Panel C: Correlation between serum CRP and SUA levels of patients with T2DM. Panel D: Correlation between serum TAC and SUA levels of patients with T2DM. Panel E: Correlation between serum MDA and SUA levels of patients with T2DM. Panel F: Correlation matrix between oxido-inflammatory biomarkers and SUA levels of patients with T2DM. Numbers represent the values of the Pearson correlation coefficient (r). CRP: c-reactive protein; IL6: interleukin 6; MDA: malondialdehyde; SUA: serum uric acid; TAC: total antioxidant capacity; TNF-α: tumor necrosis factor-alpha; T2DM: type 2 diabetes mellitus.

The association between SUA levels and serum levels of the oxido-inflammatory biomarkers among diabetic patients is presented in Figure 2.

SUA levels positively correlated with serum levels of the inflammatory biomarkers: TNF-α (Figure 2A), IL6 (Figure 2B), and CRP (Figure 2C) and the oxidative MDA (Figure 2E) (P<0.001, each)) among patients with T2DM. Additionally, a statistically significant negative correlation existed between SUA levels and serum TAC levels (Figure 2D) (P=0.010) among patients with T2DM.

**Figure 3:**
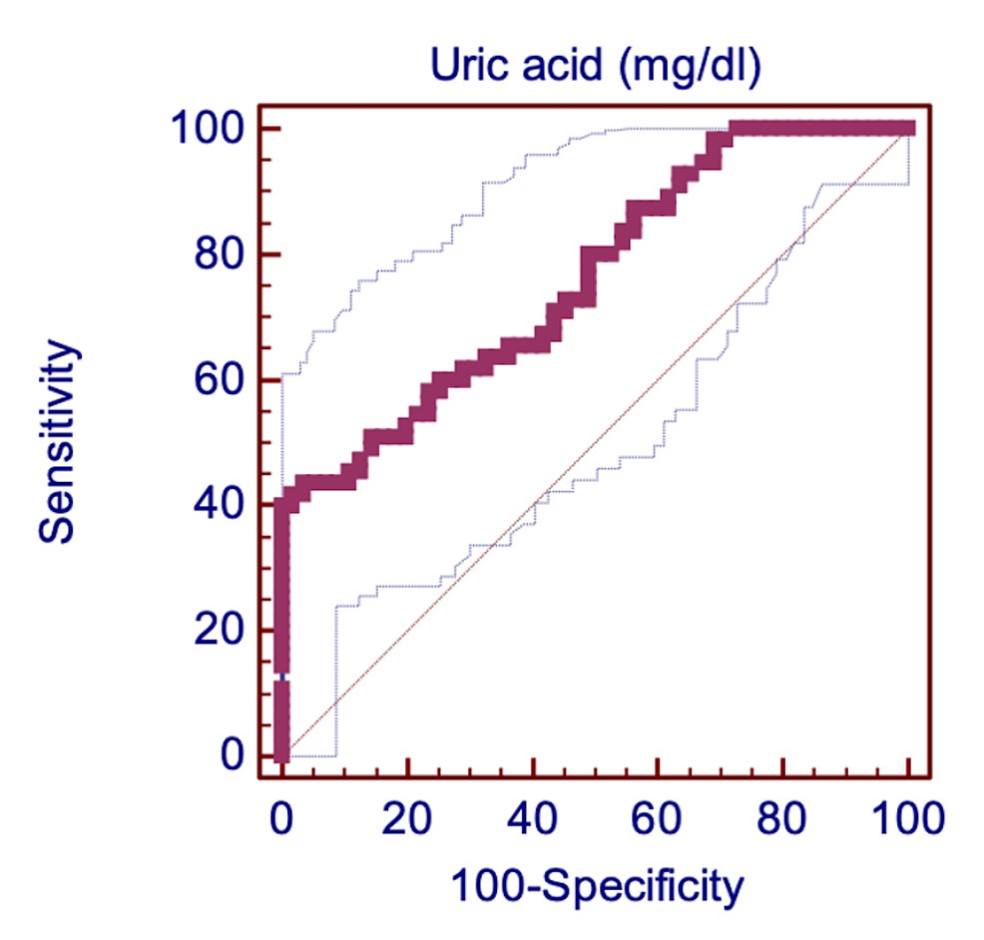
The receiver operating characteristic (ROC) curve for SUA in the prediction of CAD among patients with T2DM. The analysis demonstrated that SUA differentiates between T2DM patients with CAD and patients without CAD with an area under the curve (AUC) of 0.759 and accepted discrimination ability (95% CI: 0.67 to 0.84, P<0.001). CAD: coronary artery disease; CI: confidence interval; SUA: serum uric acid; T2DM: type 2 diabetes mellitus.

The prognostic utility of SUA among diabetic patients. The ROC curve analysis for SUA demonstrated that SUA differentiates between T2DM patients with CAD and patients without CAD with an area under the curve (AUC) of 0.759, and accepted discrimination ability (95% CI: 0.67 to 0.84, P<0.001) (Table 7 and Figure 3).

**Table 7:** Prognostic value of SUA levels in the prediction of CAD among diabetic patients. AUC: area under the curve; CAD: coronary artery disease; CI: confidence interval; SUA: serum uric acid.

CAD	Reference range	Cut-off value	Sensitivity% (95% CI)	Specificity% (95% CI)	C-statistic/AUC (95% CI)	P-value
SUA (mg/dl)	3.5-7.2	>8.35 (mg/dl)	40 (27.0-54.1)	100 (93.5-100.0)	0.759 (0.67-0.84)	<0.001

## Discussion

Diabetes mellitus has been identified as one of the four major non-communicable diseases that necessitate critical management to outline its prevalence and associated complications. Higher SUA level is associated with increased risks of all-cause and cardiovascular mortality in diabetics [10,23]. Previous studies alleged that hyperuricemia is accompanied by insulin resistance, abnormal pancreatic β-cell function, and subsequently a new onset of T2DM. These effects could be attributed to the ability of high SUA to provoke oxidative injury and inflammation within the pancreatic β-cells. Additionally, SUA also prompts inducible nitric oxide (NO) synthase (iNOS) gene expression with the resultant NO-induced β-cell dysfunction [24]. Our results showed that diabetic patients with CAD showed higher levels of SUA, inflammatory biomarkers (TNF-α, IL6, and CRP), and oxidative marker MDA, while having lower levels of serum TAC compared to healthy participants and diabetic patients without CAD. Significant positive correlations existed between SUA levels and serum levels of the inflammatory biomarkers and the oxidative MDA, while a significant negative correlation existed between SUA levels and serum TAC levels. Our results coincided with the results of Ekici et al. [17], who proved a positive association between the SUA level and the severity of CAD.

Under normal conditions, SUA has the potential to scavenge free radicals. However, reactive oxygen species are generated concurrently with the production of UA by xanthine oxidase in the case of hyperuricemia. In addition, SUA is a pro-oxidant in the atherosclerotic medium [25,26]. Our findings revealed that diabetic patients with higher SUA levels had significantly elevated serum levels of TNF-α, IL6, CRP, and MDA. In support, former studies highlighted the oxidative-inflammatory injury of UA through declining NO bioavailability leading to endothelial dysfunction [27], diminishing the anti-inflammatory adiponectin in adipose tissues [28], activating the proliferation of vascular smooth muscle cells and the production of angiotensin II [29] and initiating chronic inflammatory reactions [30].

Inflammation has a critical role in the pathogenesis of atherosclerosis. Atherosclerosis pathological events comprise fatty plaque generation, excess intimal fibrosis, smooth muscle cell proliferation, and translocation of several types of cells in response to inflammatory chains, for example, monocytes, T cells, and thrombocytes. Furthermore, oxidation of LDL-C as the first step of atherosclerosis, intensified lipoperoxidation as evidenced by excess MDA. Severe hypercholesterolemia and peroxynitrite concentration accelerate and augment atherosclerosis and vascular injury [31]. Oxygen-free radicals and uric acid control multiple intracellular signaling pathways, and variations in the involved pathways may lead to the development of atherosclerotic plaques [25].

It is well-known that vessel calcification is an indicator of atherosclerosis [32]. The earlier study by Jun and colleagues [33] elucidated the predictive value of moderate coronary calcification of SUA when added to traditional cardiovascular risk factors. Similarly, the present results validated the prognostic value of SUA in patients with T2DM in the prediction of CAD risk.

In the present study, diabetic patients with high SUA levels demonstrated significantly elevated body mass index as well as significantly higher serum TNF-α, IL6, and CRP levels. In corroboration, Stanimirovic and coworkers [34] supposed that obese individuals had higher CRP levels as adipocytes synthesize TNF-α and IL-6, which are crucial for CRP activation.

The association between uric acid and a wide range of cardiovascular diseases is witnessed not only in overt hyperuricemia (SUA >7 mg/dl in males and >6 mg/dl in females), but also in upper normal values of SUA (SUA >5.5 mg/dl) [35]. Like our findings, the risk of CAD and other risk factors (body mass index, FBG, lipid profile, and oxido-inflammatory biomarkers) were observed in diabetic patients with high (>7.56 mg/dl) and intermediate SUA (5.39-7.55 mg/dl) compared with patients with low SUA (<5.39 mg/dl).

## Conclusions

Elevated serum levels of SUA and oxido-inflammatory biomarkers are associated with an increased risk of CAD in patients with T2DM. Additionally, SUA is a simple, inexpensive, and widely available biomarker in clinical practice. SUA demonstrated prognostic utility in the prediction of the risk of CAD in patients with T2DM.
